# Normal Values of Right Ventricular Echocardiographic Parameters

**DOI:** 10.5812/cardiovascmed.14760

**Published:** 2013-10-28

**Authors:** Zahra Khajali

**Affiliations:** 1 Department of Cardiology, Rajaie Cardiovascular Medical and Research Center, Iran University of Medical Sciences, Tehran, IR Iran

**Keywords:** RV Diastolic Dysfunction, RV Mass, Ethnic Group

Right ventricle has been a forgotten chamber and for many years, just a few studies were done for evaluation of its function, especially diastolic function. In recent years, we found that right ventricle involved in patients with different diseases especially lung disease and then significant symptoms appeared. In fact, researchers found that change in size and function of right ventricle could be a sign of cardiopulmonary disease. In addition, left sided heart disease causes right sided dysfunction (1). Diastolic function of this chamber could be as important as Left ventricle diastolic function and causes diverse and important symptoms. Regarding these issues, the study about this chamber, specifically diastolic function is considerable. So, recent studies concentrated on right ventricle diastolic function in health and have been published as guidelines since 2005 and revised in 2010 (2, 3), In this issue of the journal, Shojaiefard et al. reported the normal values of right ventricular echo parameters (4). But about this study, I was wondering why authors think that diastolic function of RV might be different in diverse races and population. Although recently, researchers found different sizes and systolic function in right ventricle in various age, sex and race/ethnicity applying cardiac magnetic imaging (CMR) (5), It is logical to work initially on different sizes of chambers in our society and in fact obtain valid data related to important and common aspects like left ventricle and right ventricle, atria sizes and ventricles’ systolic function in Iranian population. besides, exercise can effect on diastolic function of chambers (6) but authors did not mention this factor in the study. Authors should have clearly explained diastolic function changes in different sex and ages. According to recent large study by Dr. Kawut et al., right ventricle mass decreased by increasing age and this change is observed more significant in men than women. By aging, right ventricle volume decreases and this change again is observed significantly in men. In addition, men had higher RV mass and larger end diastolic volume but lower RV ejection fraction. Regarding these results, diastolic function of right ventricle changes with age and sex (5). Another important sight of this study was small sample size. The studies showing the differences of size and function of cardiac chambers based on age, sex and race are performed on more than 1000 sample size. This sample size is very small even for evaluation according to sex and body surface area. Finally, I think that we should first study and survey the differences of echocardiographic data on important and common aspects (chamber sizes and systolic function) and prove these different measurements then focus on trivial issues as right ventricle diastolic function.
